# PTX3 promotes IVIG resistance-induced endothelial injury in Kawasaki disease by regulating the NF-κB pathway

**DOI:** 10.1515/biol-2022-0735

**Published:** 2023-10-24

**Authors:** Ye Sun, Lihua Liu, Ruihua Yang

**Affiliations:** Children’s Hospital of Shanxi (Women Health Center of Shanxi), No. 65, Jinxi Street, Taiyuan, Shanxi 030025, China

**Keywords:** PTX3, NF-κB pathway, IVIG resistance, Kawasaki disease, coronary artery endothelial cells

## Abstract

Intravenous immunoglobulin (IVIG) resistance leads to serious complications in Kawasaki disease (KD) with no effective treatment. This study aimed to investigate the effects of pentraxin 3 (PTX3) on human coronary artery endothelial cells (HCAECs). PTX3 levels were measured using quantitative real-time PCR (qRT-PCR), enzyme-linked immunosorbent assay, and western blotting. Cell viability was detected using the MTT assay. Biological functions were analyzed using CCK-8, EdU, flow cytometry, TUNEL, and qRT-PCR. The levels of factors of the NF-κB pathway were examined using western blotting. The results demonstrated that PTX3 expression was highest in patients and HCAECs with IVIG-resistance. Knockdown of PTX3 promoted proliferation and suppressed apoptosis and inflammation of IVIG-resistant HCAECs, whereas PTX3 overexpression produced the opposite results. Moreover, PTX3 activated the NF-κB pathway in IVIG-resistant HCAECs. A rescue study showed that PTX3 modulated biological behaviors by regulating the NF-κB pathway. Overall, our findings demonstrate that PTX3 promotes IVIG resistance-induced endothelial injury by activating the NF-κB pathway, suggesting that PTX3 may become a novel therapeutic target for patients with IVIG-resistant KD.

## Graphical abstract

**Figure:**
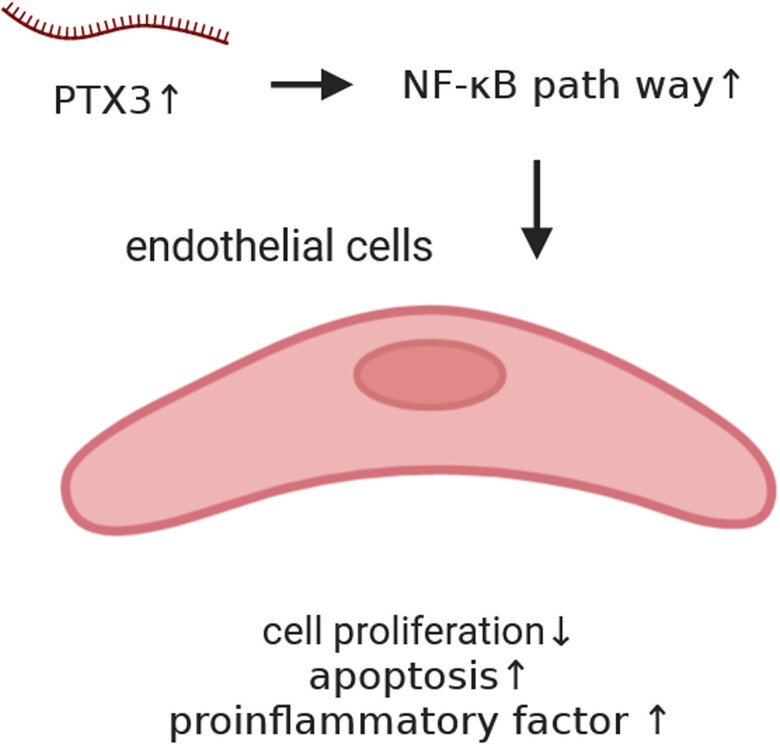

## 1 Introduction

Kawasaki disease (KD) is an acute vascular inflammation, characterized by fever, conjunctivitis, rash, lymph node enlargement, and other clinical signs, that mostly occurs in children [1]. It is an important cause of acquired heart disease in children and is prone to dramatic complications such as coronary aneurysms [2]. Administration of intravenous immunoglobulin (IVIG) is the standard treatment for KD. However, more than 10% of patients exhibit IVIG resistance [3,4]. Patients who present with IVIG resistance experience more life-threatening complications, such as KD shock syndrome and/or KD macrophage activation syndrome [5]. Unfortunately, the pathogenesis of KD and the mechanism underlying IVIG resistance remain unclear. Therefore, there is currently no reliable treatment for patients with IVIG-resistant KD [6].

Pentraxin 3 (PTX3) is one of the soluble pattern recognition molecules (PRMs) of the innate immune system [7]. PRMs mediate the humoral immunity of the host against infection and injury. PTX3 is produced by multiple cell types, such as mesenchymal, smooth muscle, and endothelial cells, under the stimulation of proinflammatory factors, microorganisms, or other agents. [8]. PTX3 has long been thought to play a crucial role in microbial resistance and inflammatory regulation [9,10]. At present, PTX3 is considered the main regulator of bone homeostasis, tissue repair, and cancer progression [11,12]. The expression of PTX3 has been detected in patients with KD, and the results showed that PTX3 is a biomarker of IVIG resistance and formation of coronary artery lesions [13]. However, the functional role of PTX3 in IVIG resistance is not understood.

This study explored the role of PTX3 in IVIG resistance by analyzing whether PTX3 affects endothelial cell functions. We speculated that PTX3 regulates cell proliferation, apoptosis, and inflammation by mediating the NF-κB pathway. The data indicated that PTX3 is a potential therapeutic target for patients with IVIG-resistant KD.

## 2 Materials and methods

### 2.1 Serum samples

A total of 20 patients with IVIG-resistant KD, 18 with IVIG-responsive KD, and 22 healthy children were enrolled in this study. Children with other diseases were excluded. Whole blood was collected from each subject and centrifuged at 1,000×*g* for 10 min to obtain serum samples, which were stored at −80°C until use. Written informed consent was signed by the guardians of all participants.


**Informed consent:** Informed consent has been obtained from all individuals included in this study.
**Ethical approval:** The research related to human use has been complied with all the relevant national regulations, institutional policies and in accordance with the tenets of the Helsinki Declaration, and has been approved by the Ethics Committee of Children’s Hospital of Shanxi (Women Health Center of Shanxi).

### 2.2 Cell culture

Human coronary artery endothelial cells (HCAECs) were purchased from Procell (Wuhan, China). The cells were cultured in a complete culture medium for HCAECs (Procell) at 37°C, 95% air, and 5% CO_2_ conditions.

To establish an IVIG resistance system, HCAECs were seeded into six-well plates and cultured until they reached 90% confluence. The cells were then incubated with RPMI-1640 medium supplemented with 5, 10, or 15% serum from patients with IVIG-resistance, IVIG responses, and healthy controls for 48 h. In addition, HCAECs were incubated with 15% serum for 0, 24, 48, and 72 h to screen induction time.

To inactivate the NF-κB pathway, 1.0 mmol/L TPCA-1 (IKK-2-specific inhibitor; MedChem Express, Monmouth Junction, USA) was added and incubated with HCAECs for 24 h.

### 2.3 Enzyme-linked immunosorbent assay (ELISA)

HCAECs culture medium was centrifuged at 3,000 rpm for 10 min, and the supernatant was collected. The concentration of PTX3 in the serum of patients and cell supernatant was measured using a human PTX3 ELISA kit (Abcam, Cambridge, USA) according to the manufacturer’s instructions.

### 2.4 Cell transfection

HCAECs were seeded into six-well plates 24 h before transfection. The cells were transfected with si-NC, si-PTX3 1#, si-PTX3 2#, empty vector, and PTX3 overexpressing vector (GenePharma, Shanghai, China) using Lipofectamine 2000 (Invitrogen, Thermo Fisher Scientific). Cell transfection was performed for 48 h. Subsequently, the IVIG resistance cell model was established.

### 2.5 Quantitative real-time PCR (qRT-PCR)

Total RNA was extracted from the cells using TRIzol reagent (Invitrogen). qRT-PCR of mRNAs was performed using a one-step RT-qPCR kit (SYBR Green) (KeyGEN, Nanjing, China) on a LightCycler 480 system (Roche, Basel, Switzerland). RNA levels were calculated using the 2^−ΔΔCt^ method. GAPDH was used as the internal control.

### 2.6 Western blotting

Total proteins were isolated from HCAECs using RIPA lysis buffer (KeyGEN). After measuring the protein concentration using a BCA kit (Beyotime, Shanghai, China), proteins were run on SDS-polyacrylamide gels and transferred to PVDF membranes. After blocking with 5% skim milk, the membranes were incubated with primary antibodies at 4°C overnight, followed by incubation with secondary antibody at 37°C for 1 h. The bands were visualized using an ECL reagent (Beyotime). GAPDH was used for normalization.

The information regarding the antibodies used is as follows: anti-PTX3 (ab125007; 1/5,000) and anti-EDN1 (ab117757; 1/3,000) were purchased from Abcam. Anti-nitric oxide (NO; 1/500) was acquired from Y-J Biological (Shanghai, China). Other antibodies were purchased from Cell Signaling Technology (Danvers, USA): anti-NFKB1 p105/p50 (#3035; 1:1,000), anti-p-NF-κB p105 (#4806; 1:1,000), anti-NF-κB p65 (#8242; 1:1,000), anti-p-p65 (#3039; 1:1,000), anti-GAPDH (#5174; 1:1,000), and the secondary antibody HRP-linked anti-rabbit IgG (#7074, 1:3,000).

### 2.7 MTT assay

HCAECs were incubated with serum for the specified time. The cells were then treated with 20 μL MTT reagent (Solarbio, Beijing, China) for 2 h. The absorbance at 490 nm was detected using an iMark microplate reader (Bio-Rad).

### 2.8 Cell counting kit-8 (CCK-8) assay

The transfected cells were cultured at 37°C for 24 h. Next, the cells were treated with 10 μL CCK-8 reagent (Solarbio) for 2 h. The absorbance at 450 nm was detected using an iMark microplate reader.

### 2.9 5-Ethynyl-20-deoxyuridine (EdU) assay

A Click-iT EdU Image kit (Invitrogen) was used to analyze cell proliferation. HCAECs were seeded into six-well plates and stained with 10 μM EdU solution for 60 min. The cells were then fixed, permeabilized, and incubated with the Click-iT^®^ reaction cocktail for 30 min. DAPI was used to stain the DNA. Photographs of the results were obtained using a fluorescence microscope (Olympus, Tokyo, Japan).

### 2.10 Flow cytometry

HCAECs were resuspended using 1× binding buffer (100 μL) in the centrifuge tubes. Annexin V-PE (5 μL) and 7-AAD staining solution (10 μL) in an Annexin V-PE/7-AAD Apoptosis Detection kit (Yeasen, Shanghai, China) were incubated with the cells for 15 min. After adding 1× binding buffer (400 μL), flow cytometry was performed within 1 h using a CytoFLEX flow cytometer (Beckman Coulter, Fullerton, USA).

### 2.11 TUNEL assay

TUNEL assay was carried out using a TUNEL Apoptosis Detection kit (Yeasen). After washing, HCAECs were fixed with paraformaldehyde for 20 min on ice. Permeabilization was performed using 0.2% Triton X-100 at 25°C for 5 min. Next, the cells were incubated with TdT reaction buffer containing Alexa Fluor 488-12-dUTP Labeling Mix at 37°C for 60 min. Finally, stained cells were visualized and photographed using fluorescence microscopy.

### 2.12 Bioinformatics analysis

The relationship between PTX3 and the NF-κB pathway was predicted using the GeneMANIA database (http://genemania.org/).

### 2.13 Statistical analysis

All experiments were repeated three times. Analysis was performed using the GraphPad Prism 8.0 software and the results are shown as the mean ± SD. The unpaired Student’s *t*-test (two groups) or one-way ANOVA (multiple groups) were used for comparisons. Differences at *P* < 0.05 indicate statistical significance.

## 3 Results

### 3.1 PTX3 is highly expressed in patients and cells with IVIG resistance

**Figure 1:**
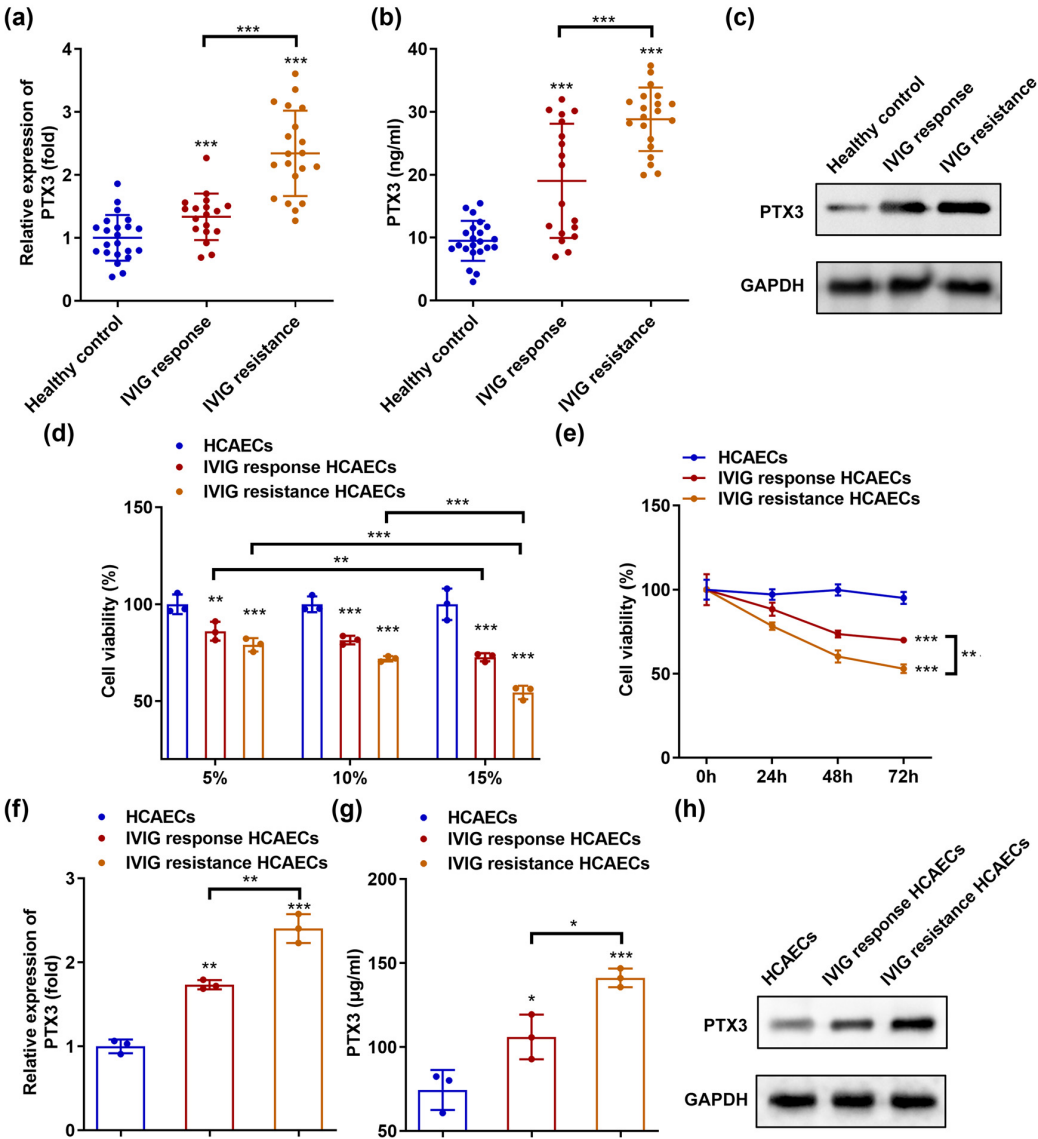
PTX3 is highly expressed in patients and cells with IVIG resistance. The levels of PTX3 were measured in the serum of healthy controls and of patients who showed IVIG responses or IVIG resistance using (a) qRT-PCR, (b) ELISA, and (c) western blotting. (d) HCAECs were incubated with 5, 10, or 15% serum for 24 h, and cell viability was evaluated using the MTT assay. (e) HCAECs were incubated with 15% serum for 0, 24, 48, and 72 h; MTT assays assessed cell viability. (f) qRT-PCR, (g) ELISA, and (h) western blotting were used to detect PTX3 levels in HCAECs, IVIG-responsive HCAECs, and IVIG-resistant HCAECs. *P < 0.05, **P < 0.01, and ***P < 0.001.

We first evaluated the levels of PTX3 in the serum samples from all participants using qRT-PCR and ELISA. PTX3 expression and concentration were significantly elevated in patients with IVIG responses compared with healthy controls. Moreover, PTX3 expression was markedly higher in patients with IVIG resistance than those with IVIG responses (Figure 1a and b). The results of western blotting were the same as those obtained using qRT-PCR or ELISA (Figure 1c). HCAECs incubated with patient serum were used to establish the cell models. Cell viability decreased in a dose-dependent manner in each model (Figure 1d). In addition, viability in IVIG-responsive and IVIG-resistant HCAECs decreased in a time-dependent manner. However, no significant differences were noted between cultures of 48 and 72 h (Figure 1e). Thus, cell models were established using 15% serum for 48 h. PTX3 levels were significantly higher in IVIG-responsive HCAECs than in HCAECs, with the highest levels observed in IVIG-resistant HCAECs (Figure 1f–h).

### 3.2 Knockdown of PTX3 inhibits IVIG resistance-induced endothelial injury

**Figure 2:**
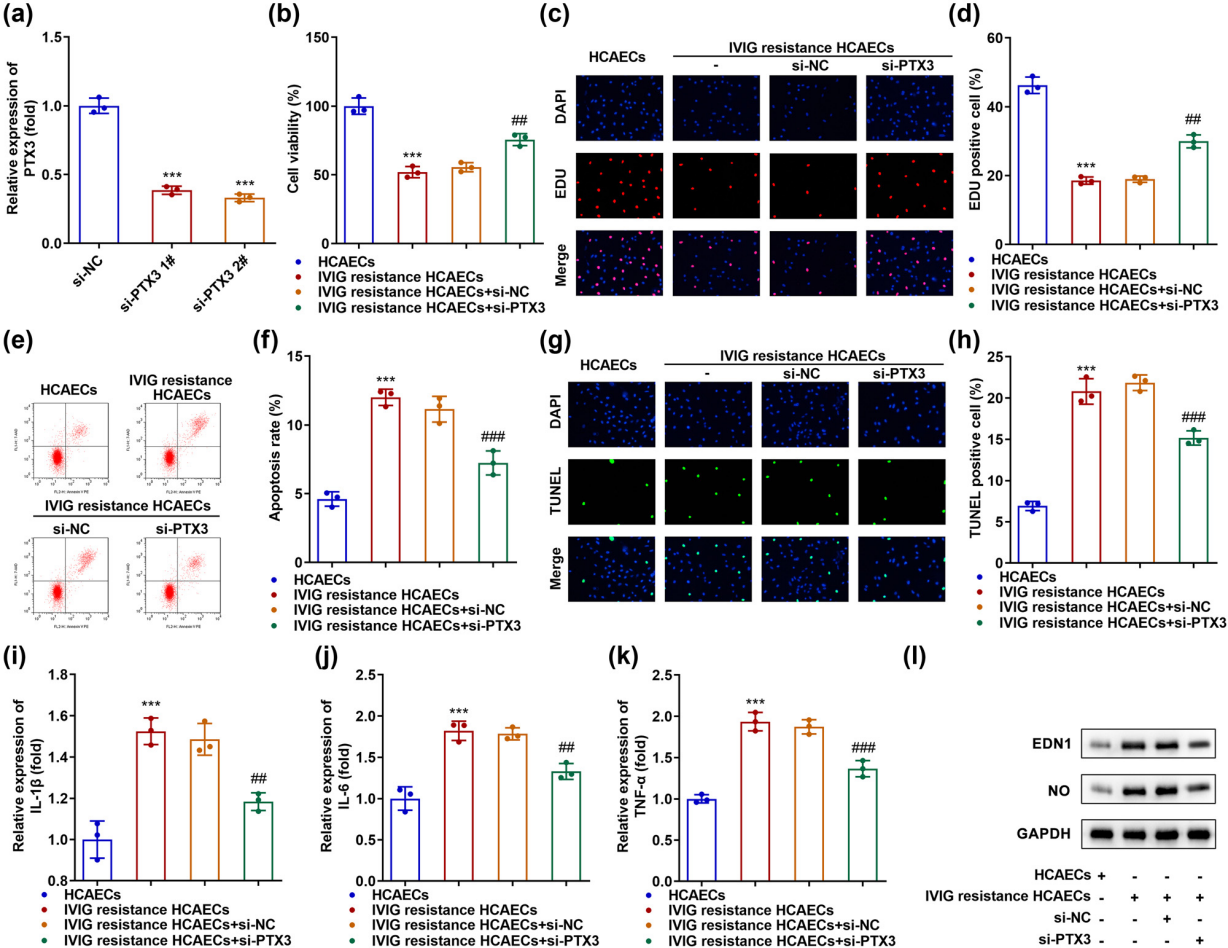
Knockdown of PTX3 inhibits IVIG resistance-induced endothelial injury. (a) Levels of PTX3 were examined using qRT-PCR in si-NC, si-PTX3 1#, and si-PTX3 2# transfected HCAECs. (b) CCK-8 and (c and d) EdU assays were used to evaluate cell proliferation. Apoptosis was evaluated using (e and f) flow cytometry and (g and h) TUNEL analysis. Inflammatory factors (i) IL-1β, (j) IL-6, and (k) TNF-α were measured using qRT-PCR. (l) Protein levels of EDN1 and NO were measured using western blotting. ***P < 0.001, ^##^ *P* < 0.01, and ^###^ *P* < 0.001.

To explore the biological function of PTX3 in IVIG-resistant HCAECs, the cells were transfected with si-NC and si-PTX3. PTX3 expression was markedly downregulated in si-PTX3 1# and si-PTX3 2# transfected cells, especially in the latter, compared with that in cells transfected with si-NC (Figure 2a). Subsequently, we found that cell proliferation was inhibited in IVIG-resistant HCAECs, while PTX3 knockdown reversed this inhibition (Figure 2b–d). Apoptosis was facilitated in IVIG-resistant HCAECs, and PTX3 knockdown inhibited IVIG resistance-induced apoptosis (Figure 2e–h). The levels of IL-1β, IL-6, and TNF-α were elevated in IVIG-resistant HCAECs, and depletion of PTX3 decreased these levels (Figure 2i–k). Moreover, the levels of endothelial injury markers, EDN1 and NO, were increased in IVIG-resistant HCAECs, and PTX3 knockdown downregulated their levels (Figure 2l). The results showed that PTX3 silencing facilitated cell proliferation and inhibited apoptosis and inflammation in IVIG-resistant HCAECs.

### 3.3 Overexpression of PTX3 promotes IVIG resistance-induced endothelial injury

**Figure 3:**
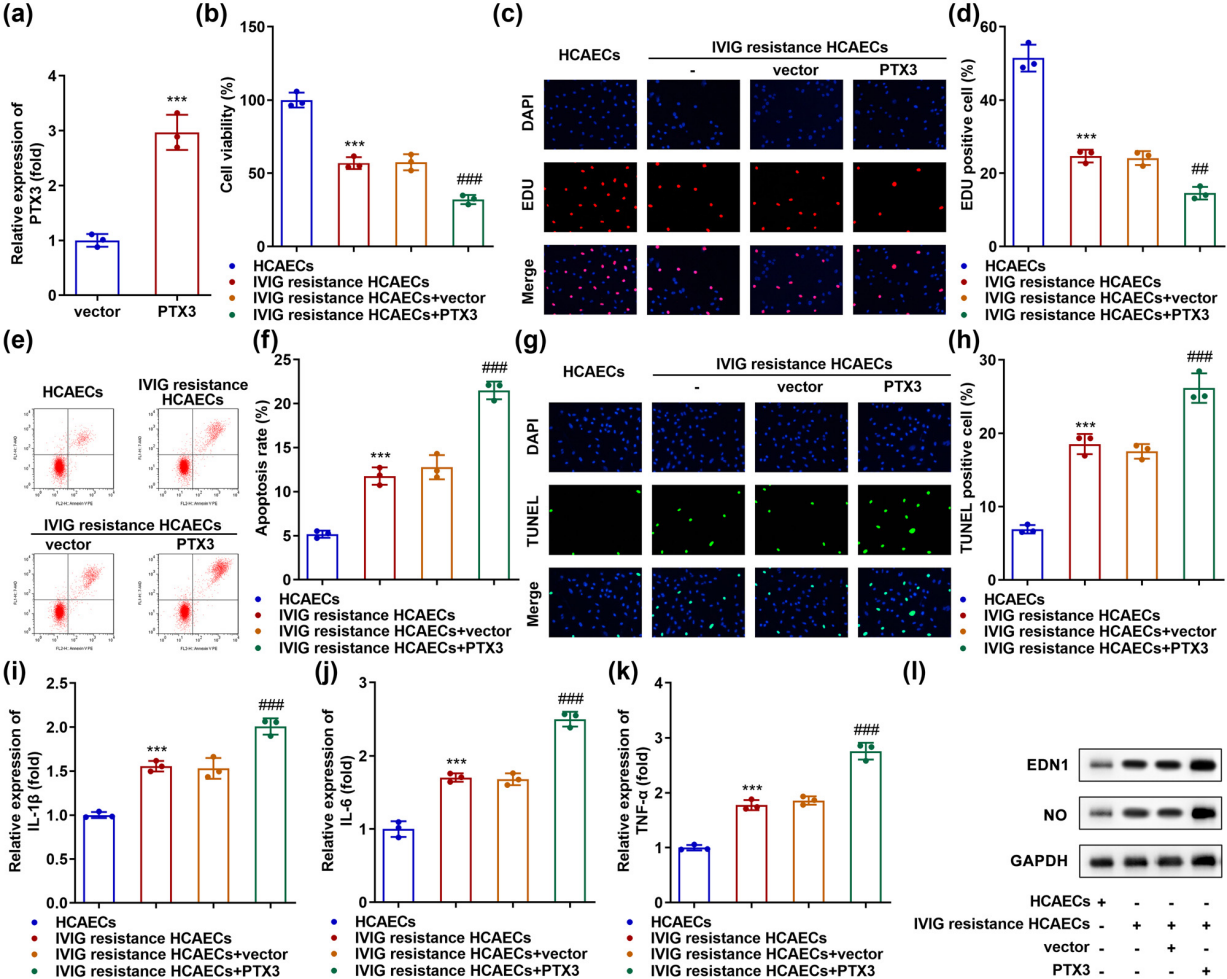
Overexpression of PTX3 promotes IVIG resistance-induced endothelial injury. (a) Levels of PTX3 were examined using qRT-PCR in the empty vector- and PTX3 overexpression vector-transfected HCAECs. (b) CCK-8 and (c and d) EdU assays were used to evaluate cell proliferation. (e and f) Flow cytometry and (g and h) TUNEL analysis evaluated apoptosis. qRT-PCR analyses were used to measure the levels of inflammatory factors (i) IL-1β, (j) IL-6, and (k) TNF-α. (l) Western blotting was performed to measure the protein levels of EDN1 and NO. ***P < 0.001, ^##^ *P* < 0.01, and ^###^ *P* < 0.001.

Following transfection with the PTX3 overexpression vector, PTX3 expression was significantly upregulated compared with that in cells transfected with the empty vector (Figure 3a). The inhibition of cell proliferation in IVIG-resistant HCAECs was further inhibited by PTX3 overexpression (Figure 3b–d). IVIG-resistant HCAECs showed higher apoptosis levels than HCAECs, and overexpression of PTX3 facilitated apoptosis in IVIG-resistant HCAECs (Figure 3e–h). IL-6, IL-1β, and TNF-α levels were upregulated in IVIG-resistant HCAECs, while overexpression of PTX3 further increased their levels (Figure 3i–k). Besides, PTX3 overexpression further elevated the protein levels of EDN1 and NO in IVIG-resistant HCAECs (Figure 3l).

### 3.4 PTX3 regulates the NF-κB pathway

**Figure 4:**
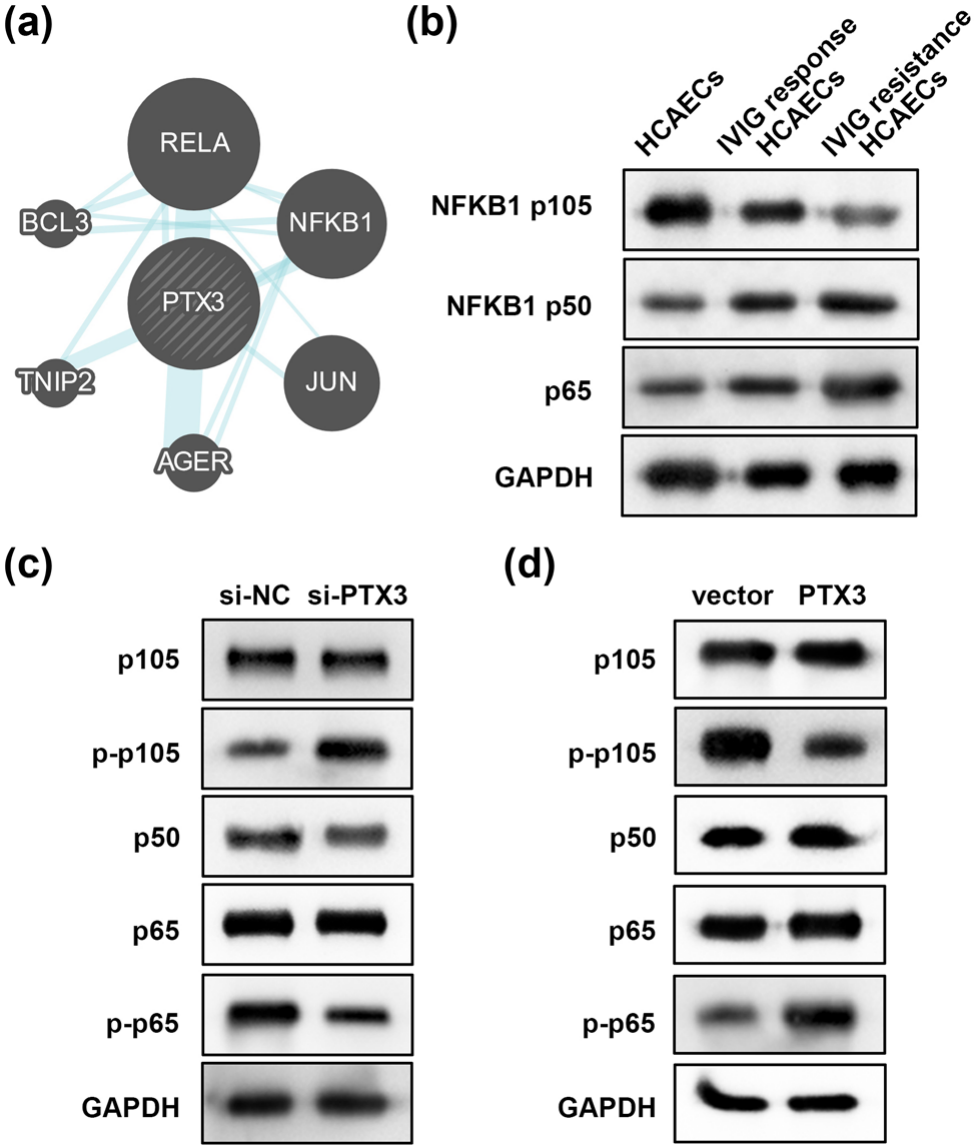
PTX3 regulates the NF-κB pathway. (a) Interaction between PTX3 and the NF-κB pathway was predicted using the GeneMANIA database. (b) Levels of NFKB1 (p105/p50) and p65 were measured using western blotting in HCAECs, IVIG-responsive HCAECs, and IVIG-resistant HCAECs. (c) Following si-PTX3 and si-NC transfection, the protein expression of p105, p-p105, p50, p-p65, and p65 was assessed using western blotting. (d) Following transfection with the PTX3 vector and empty vector, the protein expression of p105, p-p105, p50, p-p65, and p65 was assessed using western blotting.

According to the results of the bioinformatics analysis, PTX3 is associated with NFKB1 and RELA (Figure 4a). Therefore, we speculated that PTX3 regulates the NF-κB pathway. The expression levels of NFKB1 and RELA (p65) were measured in the cell model. IVIG-resistant HCAECs showed the lowest p105 expression and the highest p50 and p65 expressions (Figure 4b). Subsequently, the results of western blotting showed that knockdown of PTX3 upregulated p-p105 expression and downregulated p50 and p-p65 levels but did not influence p105 and p65 levels (Figure 4c). Conversely, overexpression of PTX3 markedly reduced p-p105 expression and increased p50 and p-p65 levels, but, similar to the previous results, did not affect p105 and p65 levels (Figure 4d). These findings suggest that PTX3 promotes the activation of the NF-κB pathway.

### 3.5 PTX3 promotes IVIG resistance-induced endothelial injury by regulating the NF-κB pathway

**Figure 5:**
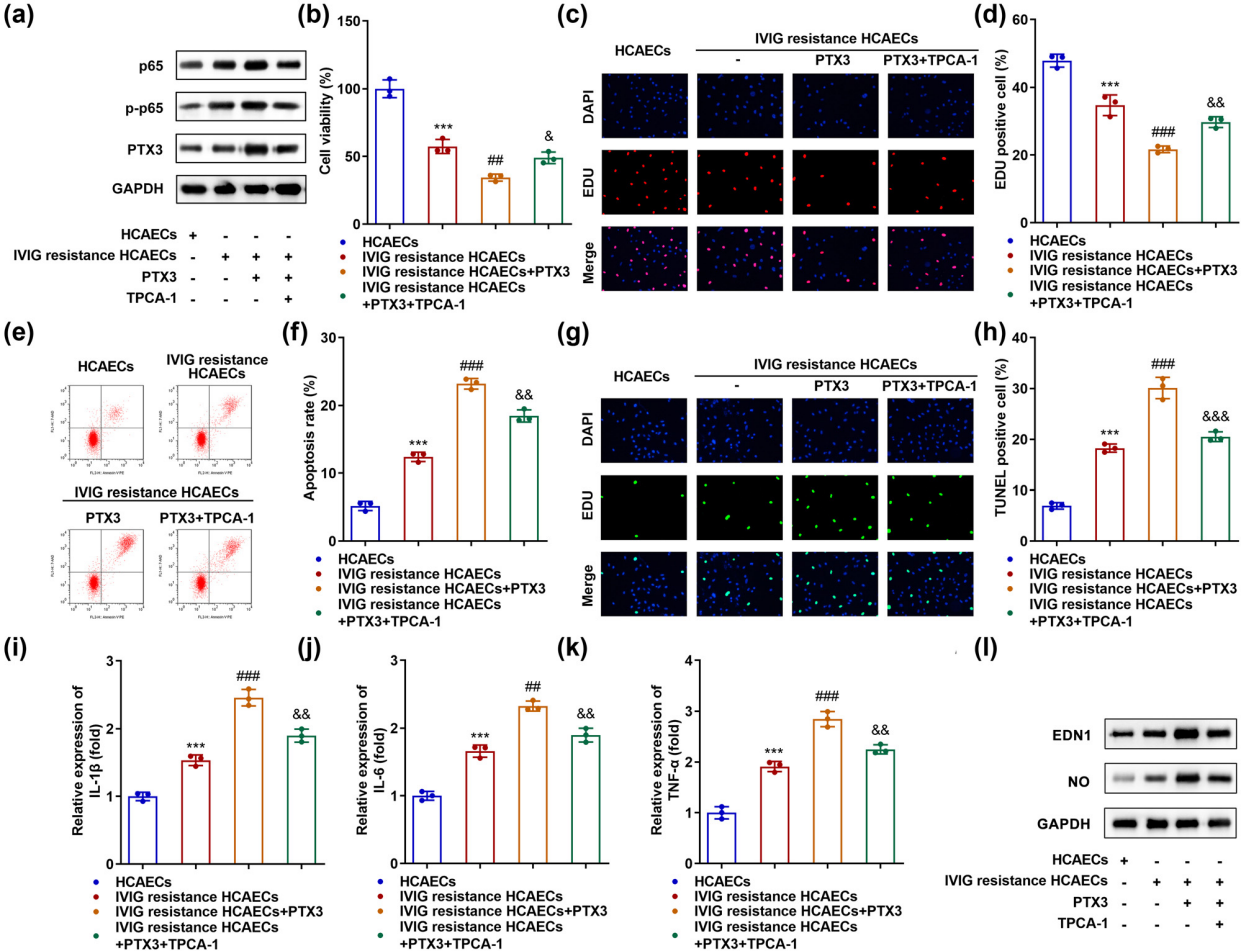
PTX3 promotes IVIG resistance-induced endothelial injury by regulating the NF-κB pathway. (a) Protein expression of p65, p-p65, and PTX3 was measured using western blotting. (b) CCK-8 and (c and d) EdU assays were used to evaluate cell proliferation. (e and f) Flow cytometry and (g and h) TUNEL analysis evaluated apoptosis. qRT-PCR measured the levels of inflammatory factors (i) IL-1β, (j) IL-6, and (k) TNF-α. (l) Western blotting was performed to measure the protein levels of EDN1 and NO. ***P < 0.001, ^##^ *P* < 0.01, and ^###^ *P* < 0.001. ^&^ *P* < 0.05, ^&&^ *P* < 0.01, and ^&&&^ *P* < 0.001.

Rescue experiments were performed to determine the function of the NF-κB pathway in IVIG resistance in HCAECs. TPCA-1 was added to inactivate the pathway. The levels of p65 increased in IVIG-resistant HCAECs and were not regulated by PTX3 but decreased by TPCA-1. The levels of both p-p65 and PTX3 increased in IVIG-resistant HCAECs, and were further elevated by PTX3 overexpression, which was abolished by TPCA-1 (Figure 5a). The inhibition of IVIG-resistant HCAEC proliferation induced by PTX3 was partly reversed by TPCA-1 treatment (Figure 5b–d). Overexpression promoted apoptosis of IVIG-resistant HCAECs, whereas TPCA-1 partly reversed this effect (Figure 5e–h). The levels of IL-6, IL-1β, and TNF-α were all elevated by PTX3 in IVIG-resistant HCAECs, while TPCA-1 rescued their elevation (Figure 5i–k). Moreover, TPCA-1 partly abrogated the levels of EDN1 and NO mediated by PTX3 in IVIG-resistant HCAECs (Figure 5l).

## 4 Discussion

KD is a vascular inflammatory disease in children characterized by congenital and adaptive immune dysregulation during acute onset. KD then induces coronary artery disease, which is caused by dysfunction of vascular remodeling and endothelial cells [14]. PTX3 is a humoral immunity-related factor that plays a dual antagonistic role in immune regulation [15]. Additionally, PTX3 is associated with numerous diseases, such as cardiovascular and bone diseases, infections, and malignancies [16–19]. Some studies have reported the levels of PTX3 in KD. PTX3 expression is higher in IVIG-resistant than in IVIG-responsive KD. Thus, it is an effective diagnostic biomarker of incomplete and IVIG-resistant KD [13]. Additionally, PTX3 expression is higher in patients who show IVIG resistance with coronary artery lesions than in those without coronary artery lesions, and is considered as a risk factor for coronary artery disease [20]. However, PTX3 is rarely detected in KD-induced cerebral vasculitis [21]. Due to the abnormal expression of PTX3, we speculated that it may act as a regulator of IVIG resistance. Herein, we confirmed that PTX3 was highly expressed in patients with IVIG resistance, consistent with the results of Kitoh et al. [13]. We then established an IVIG-resistant HCAEC model and observed that PTX3 was highly expressed in IVIG-resistant HCAECs. PTX3 is associated with endothelial dysfunction suppressing cell proliferation or reducing nitric oxide synthesis [22]. Hence, the endothelial functions of PTX3 were evaluated. Knockdown of PTX3 facilitated proliferation and suppressed apoptosis and inflammation induced by IVIG-resistant HCAECs, whereas overexpression of PTX3 produced the opposite results, indicating PTX3 promotes endothelial injury, consistent with a previous study [23]. These findings suggest that PTX3 accelerates IVIG resistance-induced endothelial injury that exacerbates patients who experience IVIG resistance. Whether endothelial cells cultured with serum containing different concentrations of PTX3 affect cellular behaviors may further confirm the effect of PTX3 on endothelial injury; this will be explored in future studies. In addition, KD-mediated immunity regulates tissue injury [24] and PTX3 plays a crucial role in tumor remodeling and repair [25]. In future experiments, we will study the effects of PTX3 on tissue injury caused by KD.

The NF-κB pathway is identified as a prototypical proinflammatory pathway, but recent studies have shown that it also has anti-inflammatory effects [26]. The P105 protein encoded by NFKB1 can be processed by the proteasome to P50, which usually binds to P65 to form a heterodimer of NF-κB, activating the classic signaling pathway and further driving the expression of proinflammatory factors [27]. The p100 NFKB2 protein, as a precursor, is processed to p52 via transcriptional activity, activating the non-classical NF-κB pathway [28]. The non-classical NF-κB signaling pathway is usually associated with B cell mutations and lymphogenesis [29,30], while the classical pathway is closely linked to cell survival, proliferation, and inflammation [31,32]. Thus, we focused on the effect of PTX3 on the classical rather than on the non-classical NF-κB signaling pathway. Previous studies have revealed that PTX3 plays an important role in the regulation of endothelial functions by mediating the NF-κB pathway in multiple diseases [23,33,34]. However, the interaction between PTX3 and the NF-κB pathway in KD, especially in IVIG-resistant KD, has not been studied. In this study, PTX3 was predicted to be associated with the NF-κB pathway. Precursor protein p105 phosphorylation leads to its degradation, promoting the release of p50. p50 and p65 form a heterodimer that migrates from the cytoplasm to the nucleus, and the phosphorylated p65 maintains the activation of the NF-κB pathway [35]. We identified that PTX3 inhibited p105 phosphorylation and promoted p65 phosphorylation, suggesting that PTX3 activates the NF-κB pathway, whereas PTX3 knockdown inactivated this pathway. These results are consistent with those of previous studies that showed that PTX3 activates the NF-κB pathway. Different from the study by Fang et al. [33], PTX3 affects the NF-κB pathway by regulating the p38 MAPK pathway; however, in this study, we did not discuss the role of the p38 pathway, which will be explored in our future work. Inactivation of the NF-κB pathway rescued the effects of PTX3 on cell proliferation, apoptosis, and inflammation. Taken together, these results indicate that PTX3 promotes endothelial injury by activating the NF-κB pathway.

In conclusion, PTX3 is highly expressed in patients and HCAECs with IVIG resistance. PTX3 inhibited the proliferation and promoted apoptosis and inflammation in IVIG-resistant HCAECs by activating the NF-κB pathway. The findings suggest that PTX3 is crucial in IVIG-resistant KD and may be a new therapeutic target for patients with IVIG-resistant KD.
